# α‐Triazolylboronic Acids: A Novel Scaffold to Target FLT3 in AML

**DOI:** 10.1002/cmdc.202400622

**Published:** 2024-11-09

**Authors:** Maria Luisa Introvigne, Lorenza Destro, Luca Mologni, Valentina Crippa, Paolo Zardi, Francesco Fini, Fabio Prati, Emilia Caselli, Alfonso Zambon

**Affiliations:** ^1^ Department of Life Sciences University of Modena and Reggio Emilia Modena Italy; ^2^ Department of Chemical and Geological Sciences University of Modena and Reggio Emilia Modena Italy; ^3^ Department of Medicine and Surgery University of Milano-Bicocca Monza Italy

**Keywords:** Acute Myeloid Leukemia, FLT3 Protein Kinase, Boron Chemical

## Abstract

The treatment of acute myeloid leukemia (AML) presents a challenge to current therapies because of the development of drug resistance. Genetic mutation of FMS‐like tyrosine kinase‐3 (FLT3) is a target of interest for AML treatment, but the use of FLT3‐targeting agents on AML patients has so far resulted in poor overall clinical outcomes.^[1]^ The incorporation of the boronic group in a drug scaffold could enhance the bioavailability and pharmacokinetic profile of conventional anticancer chemotypes. Boronic acids represent an intriguing and unexplored class of compounds in the context of AML, and they are only scantly reported as inhibitors of protein kinases. We identified α‐triazolylboronic acids as a novel chemotype for targeting FLT3 by screening a library of structurally heterogeneous in‐house boronic acids. Selected compounds show low micromolar activities on enzymatic and cellular assays, selectivity against control cell lines and a recurring binding mode in *in‐silico* studies. Furthermore, control analogues synthesized *ad hoc* and lacking the boronic acid are inactive, confirming that this group is essential for the activity of the series. All together, these results suggest α‐triazolylboronic acids could be a promising novel chemotype for FLT3 inhibition, laying the ground for the design of further compounds.

## Graphical abstract

Boronic acids represent an intriguing and unexplored class of compounds in the context of acute myeloid leukemia (AML), and they are only scantly reported as inhibitors of protein kinases. The screening of an in‐house library of boronic compounds identified α‐triazolylboronic acids as a novel chemotype for targeting FLT3 in AML. Selected compounds show low micromolar activities on enzymatic and cellular assays, selectivity against control cell lines and a recurring binding mode in *in‐silico* studies.

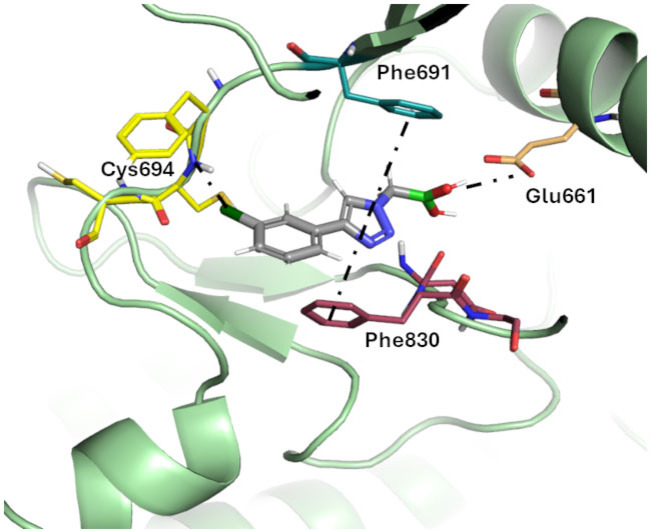

## Introduction

Acute myeloid leukemia (AML) is a form of blood cancer that originates from immature white blood cells, within the bone marrow. AML is the most prevalent form of acute leukemia in adults, constituting approximately 80 % of cases. In the USA, the incidence of AML ranges from three to five cases per 100,000 population, with an estimated 21450 new cases diagnosed in 2019 alone, resulting in over 10,000 deaths.^[2]^ AML primarily affects older adults, with the incidence increasing from around 1.3 per 100,000 population in patients less than 65 years old to 12.2 cases per 100,000 population in those over 65 years in the UK.^[3]^

AML is a treatment‐resistant disease that poses significant challenges in clinical management. Among the various genetic mutations identified in AML, mutations in the FMS‐like tyrosine kinase 3 (FLT3) gene hold particular importance. These mutations occur in approximately 30 % of all AML cases, with the internal tandem duplication (ITD) being the most prevalent type, accounting for about 25 % of all cases and the rest being mutations in the tyrosine kinase domain (TKD). Despite efforts to develop FLT3 inhibitors, the emergence of secondary TKD mutations, such as FLT3/D835Y and FLT3/F691L limited the effectiveness of FLT3‐targeting therapies on AML patients, resulting in poor overall clinical outcomes. These acquired mutations cause resistance to current FLT3 inhibitors. There is thus an area of unmet medical need in AML treatment, and there is a strong rationale in identifying novel chemotypes for FLT3 inhibitors that could act as second‐line therapies.^[1]^

The use of boron in drug design is recent since it was considered toxic because of its high reactivity as an electrophile. In its stable compounds, boron forms three bonds and has an empty *p* orbital that can react with a variety of nucleophiles to form reversible covalent bonds. However, boron is present in nature in different kind of fruit and vegetables and few compounds in nature contain boron in their structure, such as the peptidic antibiotics, borophycin, aplasmomycin, tartolon B and boromycin.^[4]^ One of boron most common compounds, boric acid, is used as ant poison but has a low human toxicity with a LD_50_ of 2660 mg/kg which is very close to that of table salt (NaCl‐LD_50_ 3000 mg/kg).^[5]^ In fact, boric acid is a component of eye wash and vaginal creams and is used as a buffer for biological assays. Recent studies which analyzed the toxicity of boronated compounds for inhalation, skin and oral exposure, and also its genotoxicity and carcinogenicity, demonstrated that boronated compounds are to be considered not intrinsically toxic. As a consequence, in recent years, boron‐containing compounds have emerged as a new and interesting field of research in medicinal chemistry.^[6]^

**Figure 1:**
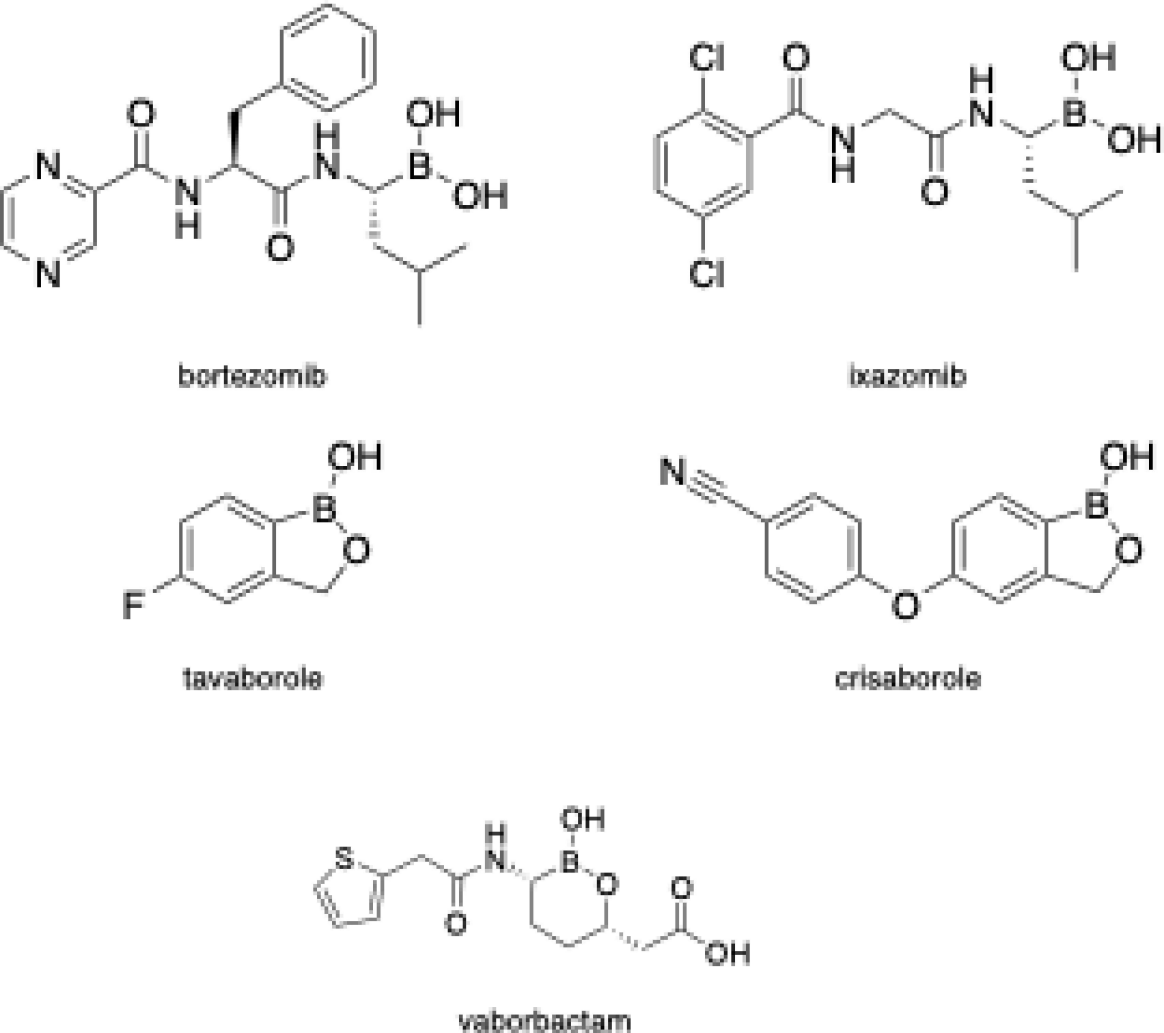
Boronic acids on the market.

The possibility to incorporate boron into drugs, particularly as boronic acids or their cyclic esters, has recently drawn considerable attention for several attractive physicochemical properties, such as low lipophilicity, water solubility, and stability. Five recently approved boron‐containing drugs comprise the boronic acid moiety or cyclic boronate (Figure 1): bortezomib (Velcade®)[^7^, ^8^] and its oral version ixazomib (Ninlaro®)^[9]^ have been approved for treatment of multilple myeloma; Tavaborole (Kerydin®) is an anti‐fungal agen;t^[10]^ Crisaborole (Eucrisa®) is on the market for the treatment of eczema and atopic dermatitis^[11]^ and Vaborbactam (in combination with meropenem in Vabomere®) for the treatment of resistant bacterial infections.^[12]^

Other molecules are under investigation in clinical and preclinical studies. Limiting to anti‐cancer agents, boronic compounds are under investigation as dipeptidyl peptidase 8 and 9 (DPP8/9) inhibitors,^[13]^ serine proteases involved in the development of different tumors and deregulated immune responses and as Histone deacetylase inhibitors. Histone deacetylase I is overexpressed in different cancer cell lines and, in particular, in multiple myeloma cells where its enhanced presence causes resistance to bortezomib.[^14^, ^15^] A boronic acid moiety is also used to improve the PK/PD profile of some anti‐cancer drugs, such as fulvestrant, for the treatment of breast cancer,^[16]^ combretastatin A‐4^[17]^ and chalcones.^[18]^ Finally, other studies are under development for an interesting application of boronic acids as anti‐cancer pro‐drugs: the reactive oxygen species (ROS), present in higher amount in cancer cells than healthy cells, oxidize boronic acids and their esters to the corresponding alcohols and boric acid, not toxic for human cells. Therefore, the replacement of a hydroxyl group with a boronic acid in a drug drives the selective release of the active moiety in the target cancer cells.^[19]^

Thus far, boronic acids have received limited attention as protein kinase inhibitors (PKIs), with few reported cases that introduce the boronic group on known structures, such as crizotinib^[20]^ and fasudil^[21]^ or the benzoimidazoles^[22]^ and pyrazolo‐quinoline.^[23]^ The boronic analogue of crizotinib is a case of boronic prodrug, to be activated by ROS species in the site of action, whereas for the fasudil derivative the boronic acid is introduced to obtain a bisubstrate‐like inhibitor of protein kinase A (PKA). Benzimidazoles with a boronic acid substituent are patented as phosphatidyl inositol 3‐kinase (PI3 K) inhibitors.^[22]^ Pyrazolo‐quinoline containing a boronic acid moiety have been investigated as inhibitors of Rho‐associated protein kinases (ROCKs), serine‐threonine kinases involved in cell proliferation, cytokinesis, apoptosis and autophagy.^[23]^

There is then an interest in the identification of novel boron‐containing chemotypes for the inhibition of protein kinases, that could potentially address significant medical needs in an unexplored intellectual property landscape.

One of the recurring interactions of the known PKIs is the binding of a polar group (e. g. amide or urea) to the conserved salt bridge between a Lys in the β3 loop and an Glu in the αC helix of the kinase. Boronic acids can act as isosteres of both the amide and the urea group, and thus boronic compounds could potentially exploit this interaction. Our group has been active for years in the development of boronic drugs as antibacterial agents, synthesizing a wide range of boronic compounds covering a large chemical space. Here we present the screening of a part of our in‐house library of boronic compounds as FLT3 inhibitors, which led to the identification of α‐triazolylboronic acid as a novel selective chemotype for the inhibition of FLT3 kinase and FLT3‐driven cells.

## Results and Discussion

### Selection and Screening of In‐House Boronic Compounds

**Scheme 1:**
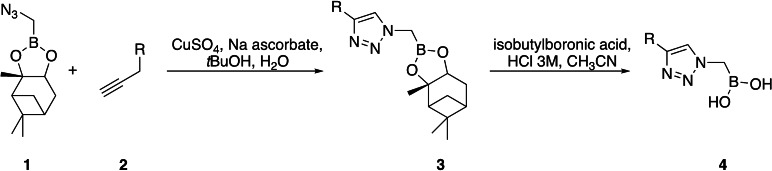
General synthesis of α‐triazolylboronic acids.

Initially, a library of sixty in‐house boronic compounds was screened for enzymatic activity against FLT3. The compounds were chosen within four classes, to allow for the maximum structural diversity: α‐amidoboronic acids (Series 1, Table 1), β‐triazolylboronic acids (Series 2), α‐sulfonamidoboronic acids (Series 3) and α‐triazolylboronic acids (Series 4). These compounds were selected from a larger library of boronic acids originally developed as Boronic Acid Transition State Inhibitors (BATSIs), known reversible covalent inhibitors of Serine β‐lactamases for the treatment of resistant bacterial infections.[^24^, ^25^, ^26^, ^27^, ^28^, ^29^, ^30^] The design of these different series of compounds was based on a biomimetic approach, decorating the boronic acid with the chemical groups of the natural substrate of the β‐lactamases, *i.e* β‐lactam antibiotics. In Series 1 and 2 the amide side chain typical of penicillin/cephalosporins was conserved,[^31^, ^32^, ^33^] while in Series 3 and 4 amide moiety was replaced by its bioisosteric sulfonamide or 1,2,3‐triazole. In our previous studies, the sulfonamide acts as amide bioisostere by retaining two of the canonical interactions in the amide binding site, while manifesting a different SAR.[^28^, ^29^, ^34^] Interestingly, also α‐triazolylboronic acids conserved the typical interactions in the amide binding site, once more highlighting triazoles as nonclassical amide bioisosters with a similar planarity, size, dipole moment, and hydrogen bonding capabilities. 1,2,3‐Triazoles 1,4‐disubstituted are synthetically accessible through a Huisgen‐type click‐chemistry reaction between azidomethaneboronate and an alkyne in a 1−3‐dipolar Cu‐catalyzed azide−alkyne cycloaddition (CuAAC), which occurs in mild conditions, with inexpensive reagents, high versatility and rapid purification of the products (Scheme 1, Experimental Section).[^35^, ^36^, ^37^, ^38^]

**Table 1:** Structure and activity against FLT3 of compounds from Series 1–4 (IC_50_, mean±SEM [μM]).

	Structure	Cmp	R_1_	R_2_	FLT3 Kinase Activity
Series 1	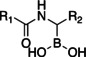	**1 a**	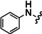	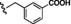	>100
		**1 b**	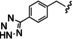	−H	>100
		**1 c**	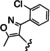	−H	>100
		**1 d**	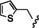	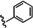	>100
		**1 e**	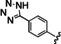	−H	>100
		**1 f**	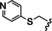	−H	>100
		**1 g**	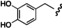	‐H	>100
Series 2	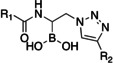	**2 a**	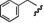	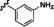	>100
		**2 b**	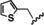	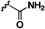	>100
		**2 c**	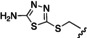	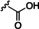	>100
		**2 d**	−CH_3_	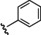	>100
		**2 e**	−CH_3_	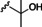	>100
Series 3	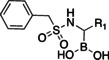	**3 a**	−H	/	>100
		**3 b**	−CH(CH_3_)_2_	/	>100
		**3 c**	−CH_3_	/	>100
Series 4	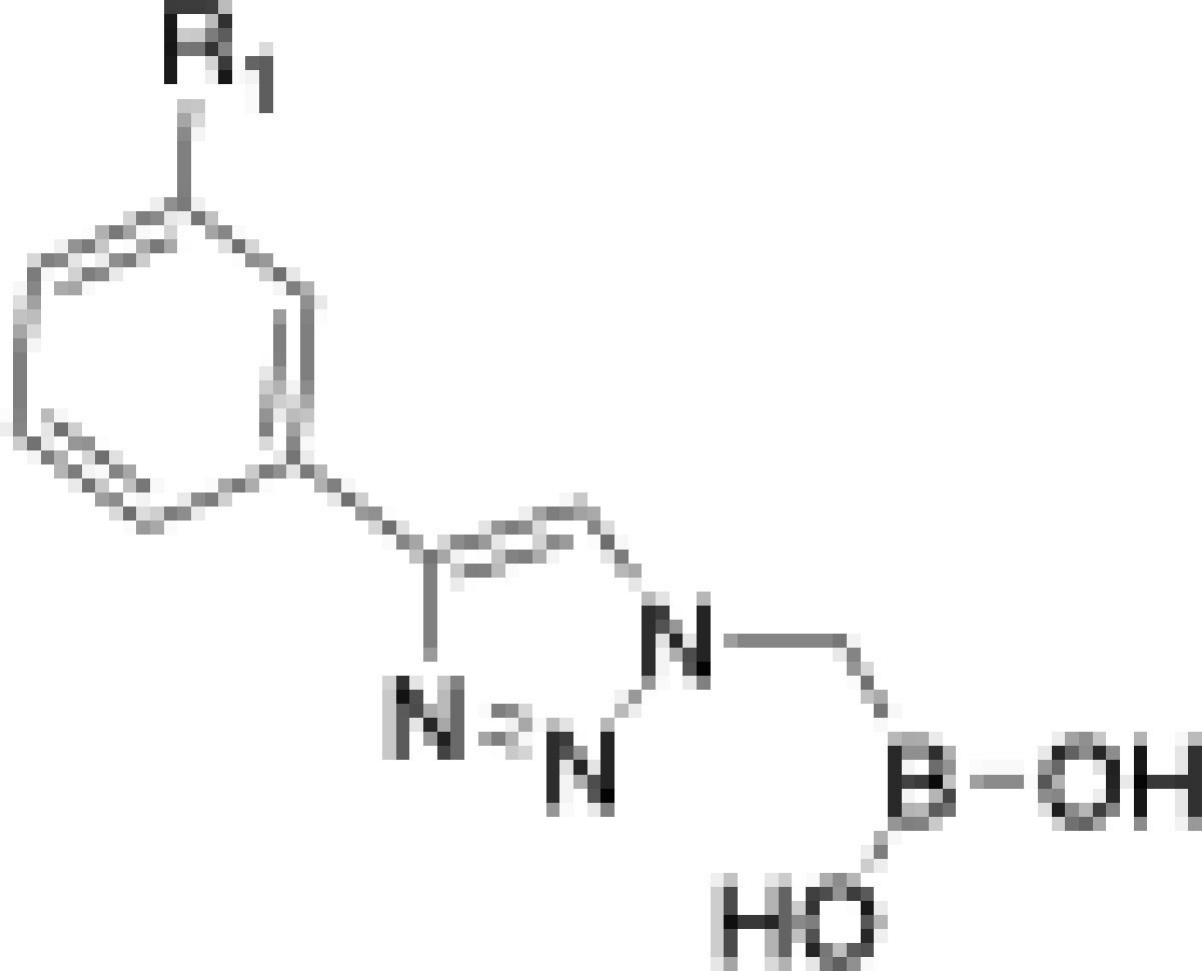	**4 a**	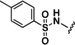	/	18±1
		**4 b**	−CONH_2_	/	>100
		**4 c**	−COOH	/	>100
		**4 d**	−CH_3_	/	25±2
		**4 e**	−NH_3_^+^CF_3_COO^−^	/	6.7±1.7
		**4 f**	−SO_2_NH_2_	/	>100
		**4 g**	−Cl	/	18±5

Selected compounds from the four series of BATSI were tested for a first screening as inhibitors of FLT3; the results are summarized in Table 1. Series1–3 did not show any activity for FLT3, with all the IC_50_ values over 100 μM; however, some α‐triazolylboronic acids form **Series 4** did show good inhibition of FLT3, with IC_50_ in the low micromolar range (IC_50_ between 5 and 30 μM). This series of compounds was further investigated by carrying out *in silico* studies to elucidate their putative binding mode and profiling their activity against FLT3‐ITD‐driven and control cell lines.

### *In‐Silico* Investigation on the Binding Mode of α‐Triazolylboronic Acids

**Figure 2:**
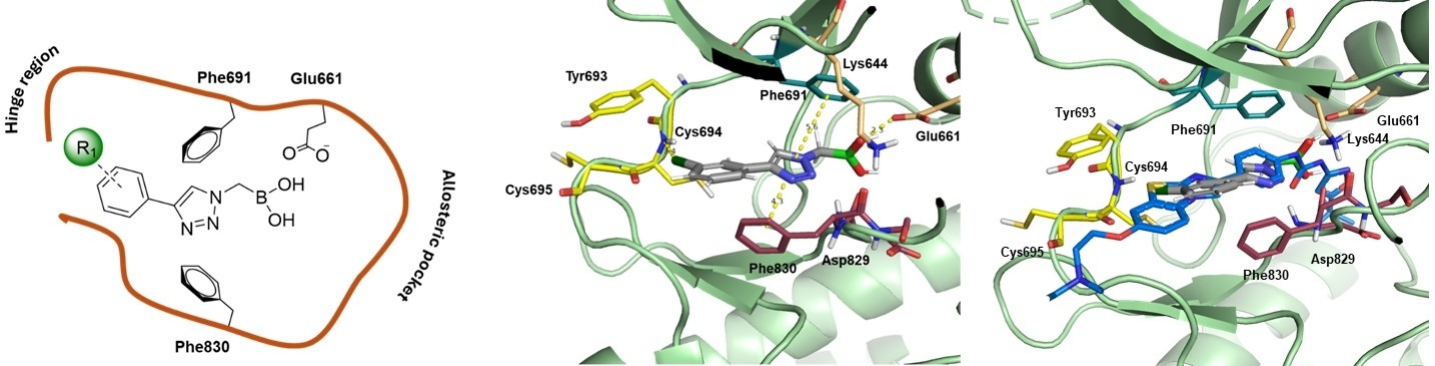
(A) Putative binding mode of α‐triazolylboronic acids on FLT3 (B) pose of boronic compound 4 g in the active site of FLT3: the boronic group of 4 g interacts with the Lys644‐Glu661 salt bridge (orange), the central aromatic ring forms π stacking with gatekeeper Phe 691 (blue) and DFG's Phe 830 (red), and the terminal ring elaborates towards the hinge region (yellow)(C) the pose of 4 g (gray) is superposable to that of quizartinib (blue).

The α‐triazolylboronic acids from **Series 4** were docked using the Glide algorithm on the available co‐crystal structure of FLT3 with quizartinib (PDB 4RT7) to gain clues on the putative mode of action of **Series 4** as ligands of the ATP binding site of the protein. These compounds showed a recurring binding mode with consistent interactions within the ATP binding site, exemplified in Figure 2A−B for **4 g**, and observed for all active compounds (**SI** Figure 1). The binding mode of the series closely resembles that of quizartinib into the ATP binding pocket. Specifically, the boronic group overlaps the urea moiety of quizartinib, and the inhibitor extends along the ATP pocket by positioning the triazole ring like the phenyl ring of quizartinib, leaving the back cleft of the protein empty (Figure 1 **C**). The compounds thus seem to bind to FLT3 as type IIB inhibitors, as already reported for other ligands of FLT3.[^39^, ^40^]

Specifically, the binding mode is characterized by robust H‐bonding between the boronic group and the salt bridge of FLT3 (Glu661‐Lys644), and a strong edge‐to‐face π‐stacking between the triazole ring and the gatekeeper Phe691 and DFG's Phe830 (Figure 1 **)**. Edge‐to‐face interaction is the preferred interface between nonsequential aromatic residues inside proteins.^[41]^ This is a recurring feature observable in both type I and type II kinase inhibitors co‐crystallized with FLT3.[^42^, ^43^, ^44^, ^45^] Finally, the substituent of the triazole ring elaborates towards the hinge region, driving the formation of possible interactions with Cys694‐Cys695. This binding mode provides a possible explanation for the lack of activity of Series 1–3. Indeed, Series 1 does not present an aromatic group able to form the crucial π‐stacking interactions with Phe 691 and Phe 830. On the other hand, Series 2 and 3 further elaborate the scaffold with a second substitution on the methylene group, clashing with the pocket of the enzyme. This observation may explain the failure to obtain a consistent binding pose for Series 1–3 on PDB 4RT7.

### Profiling of Selected of α‐Triazolylboronic Acids on FLT3‐Driven Cell Lines

Starting from the results obtained in the first screening, a second selection of α‐triazolyl boronic acids (Table 2) was accomplished and compounds were evaluated for their enzymatic inhibition. These compounds were profiled for cellular activity against FLT3+ cell lines MV‐4‐11 and MOLM‐14, both carrying the FLT3‐ITD mutation, using ponatinib as reference compound. Based on structural diversity of the substituent in position 4 of the triazole, these compounds were classified in 5 subseries characterized by the presence of aromatic (**4 a–g**), heteroaromatic (**4 h–j**‐sulfonamidomethyl (**4 k–p**), phenoxymethyl (**4 q–s**) and amido/aminomethyl (**4 t–v**) groups, plus the ester **4 w**. When tested against the recombinant FLT3 enzyme (Table 2), compound **4 t** with a benzylamido side chain bound to the triazole ring demonstrated the best inhibition with IC_50_ in the low‐micromolar range (3.2 μM); interestingly, the switch from nitrogen to oxygen (**4 t** *vs* **4 w**) is detrimental for enzymatic activity (3.2 μM *vs* >100 μM) but confers cellular penetration (>100 *vs* 1.4 μM). The change in enzymatic activity could be explained by the formation of a strong H‐bond of the NH group of **4 t** with a water molecule within the active site, which is not observed for **4 w** (SI Figure 2). In contrast, the increase in **4 w** cell activity could be related to off‐target effects combined with increased permeability or reduced efflux due to the presence of fewer H‐bond donors in the scaffold.[^46^, ^47^] The methylene bridge position of the aminomethyl series seems to tolerate only the benzylamido substitution, as both amide **4 v** and aromatic amine **4 u** lose enzymatic activity.

**Table 2:** Inhibition of FLT3 activity: enzymatic assay and cellular assays using MV‐4‐11 and MOLM‐14 cell lines (IC_50_, mean±SEM [μM]). Inhibition in MOLM‐14 cells was tested only when the result in MV‐4‐11 cells was different from >100 μM. Ponatinib was used as a reference compound.

	Cmp	R	FLT3 Kinase	MV‐4‐11	MOLM‐14
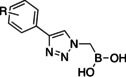	**4 a**	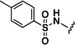	18±1	14±0.2	5.8±0.7
	**4 b**	3‐CONH_2_	>100	>100	
	**4 c**	3‐COOH	>100	>100	
	**4 d**	3‐CH_3_	25±2	11±1	3.0±0.5
	**4 e**	3‐NH_3_^+^CF_3_COO^−^	6.7±1.7	14±3	11±1
	**4 f**	3‐SO_2_NH_2_	>100	>100	
	**4 g**	3‐Cl	18±5	2.7±0.9	0.68±0.08
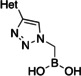	**4 h**	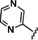	>100	>100	
	**4 i**	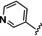	>100	>100	
	**4 j**	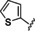	76±13	100	11±2
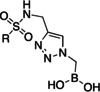	**4 k**	−CH_2_CF_3_	5.8±0.9	>100	
	**4 l**	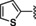	25±5	>100	
	**4 m**	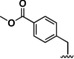	>100	>100	
	**4 n**	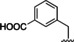	>100	>100	
	**4 o**	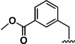	65±11	>100	
	**4 p**	−CH_3_	42±5	>100	
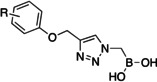	**4 q**	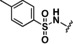	18±3	10±1	4.3±0.5
	**4 r**	−H	>100	1.1±0.1	0.16±0.01
	**4 s**	−OCH_3_	>100	6.1±1.4	14±7
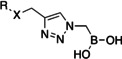	**4 t**	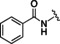	3.2±0.4	>100	
	**4 u**	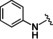	>100	7.6±3.9	8.7±1.4
	**4 v**	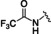	>100	>100	
	**4 w**	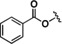	>100	1.4±0.3	0.35±0.01
**ponatinib**	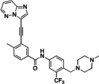		0.033±0.001	0.007±0.002	0.001±0.0001

**Figure 3:**
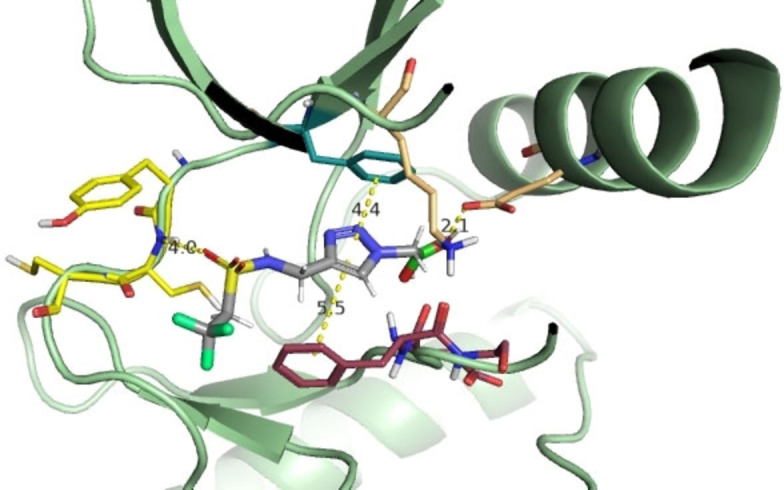
Docking of **4 k** on FLT3 (PDB 4RT7): the sulfonammido group of **4 k** forms an H bond with Cys694 of the hinge region, increasing the affinity of the subseries for FLT3.

Compounds **4 k** and **4 e** showed inhibition in the same range (IC_50_=5.8 μM and 6.7 μM). **4 k** contains a sulfonamide moiety which seems to improve the activity also in other compounds, such as **4 l** (IC_50_=25 μM), **4 o** (IC_50_=65 μM), **4 p** (IC_50_=42 μM), **4 q** (IC_50_=18 μM) and **4 a** (IC_50_=18 μM). This agrees with the postulated binding mode: the polar sulfonamide group can form H bonds with the hinge region thus enhancing the affinity of the scaffold for FLT3 (Figure 3).

Cellular assays were carried out on MV‐4‐11 cells, driven by the FLT3‐ITD mutation. Compounds active against MV‐4‐11 cells, were further tested against MOLM‐14 cells, which carry the same mutation. The compounds of the methylene‐sulfonamido subseries lost their activity in cell assays, while compounds in the aromatic and phenoxymethyl series (**4 k–p**) showed the best cellular activities. Indeed, compounds **4 a**, **4 d**, **4 e**, **4 g** and **4 q** have cellular potency, in line with their enzymatic activity, with good results on both MV‐4‐11 and MOLM‐14 cell lines. The presence of an aromatic ring bound to the triazole seems to improve the activity of boronic compounds both in enzymatic and cellular assays. Interestingly, in addition to **4 w**, compounds **4 r** and **4 s** also do not inhibit FLT3 but show significant cellular activity, confirming the ability of the series to cross the cell membrane and suggesting possible off‐target effects for these compounds. Compound **4 g** with a meta‐chloro substituent showed the best cellular activity overall, with an IC_50_ in the low micromolar range in MV‐4‐11 cells (IC_50_=2.7 μM) and in the nanomolar range in MOLM‐14 cells (IC_50_=0.68 μM). These five compounds were selected to be tested for selectivity using HL60 as control cell line.

### Selectivity Against Control Cell Line

Compounds **4 a**, **4 d**, **4 e**, **4 g** and **4 q** were among the best inhibitors in enzymatic assays and confirmed their activity also in cellular tests on FLT3‐driven MV‐4‐11 and on MOLM‐14 cell lines. To confirm their cellular mechanism of action, we assayed their cytotoxicity against FLT3‐wild type HL60 cells. Pleasingly, all compounds were selective towards FLT3‐driven cells, with only compounds **4 d** and **4 g** showing moderate inhibition of HL60 (Table 3), suggesting an on‐target mode of action in MV‐4‐11 and MOLM‐14 cells.

**Table 3:** Evaluation on HL60 cells and selectivity on selected compounds against FLT3‐driven cell lines (IC_50_, mean±SEM [μM]).

Cmp	Structure	MV‐4‐11	MOLM‐14	HL60
**4 a**	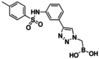	14±0.2	5.8±0.7	>100
**4 d**	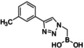	11±1	3.0±0.5	32±4
**4 e**	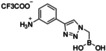	14±3	11±1	>100
**4 g**	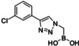	2.7±0.9	0.68±0.08	15±1
**4 q**	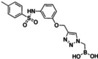	10±1	4.3±0.5	>100
**Ponatinib**	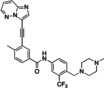	0.007±0.002	0.001±0.0001	

### Role of the Boronic Moiety

**Scheme 2:**
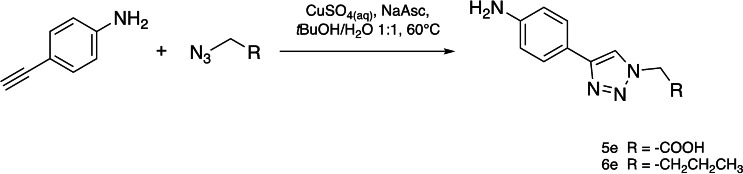
Synthesis of control compounds **5 e** and **6 e** compounds.

In order to validate the role of the boronic group on binding, we synthesized control analogues of compound **4 e** replacing the boronic group with a carboxyl group (**5 e**) and an aliphatic chain (**6 e**) by CuAAC (Scheme 2, Experimental section). Compounds **5 e** and **6 e** lost activity in all the assays (Table 4), confirming that the presence of the boronic group is essential to the pharmacophore.

**Table 4:** Tests of compounds **5 e** and **6 e** (IC_50_, mean±SEM [μM]).

Cmp	Structure	MV‐4‐11	MOLM‐14	HL60
**5 e**	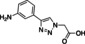	>100	>100	>100
**6 e**	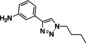	>100	>100	>100

## Conclusions

AML is a treatment‐resistant disease difficult to treat with the current available therapies. FLT3 has been proposed as a target for the treatment of AML, but so far single‐agent FLT3 inhibitor therapy has met limited clinical efficacy. There is thus a strong medical need towards the development of more selective and potent inhibitors.

Boronic acids represent an intriguing and unexplored class of compounds in the context of AML and only few examples report boronic acids as PKI, whereas the incorporation of the boronic group was demonstrated to enhance the bioavailability and pharmacokinetic profile of other conventional anticancer drugs.

We screened a selection of structurally diverse available in‐house boronic acids. Enzymatic and cellular assays lead to the identification of α‐triazolylboronic acids as a promising scaffold for FLT3 inhibition, showing a recurring binding mode in docking studies and presenting potencies in the low micromolar range in both cellular and enzymatic assays and selectivity against control cell lines. Preliminary SAR indicates that the presence of a sulfonamide moiety can improve enzymatic activity of the scaffold but is detrimental to cellular potency. Compounds from the aromatics and phenoxymethyl series combine good enzymatic activity with comparable potency on FLT3‐driven MV‐4‐11 and MOLM‐14 cell lines. Importantly, control compounds synthesized *ad‐hoc* indicate that the activity of the scaffold is linked to the presence of the boronic acid moiety. The best inhibitor in the series is **4 g**, with an IC_50_ in the low micromolar range in MV‐4‐11 cells (IC_50_=2.7 μM) and in the nanomolar range in MOLM‐14 cells (IC_50_=0.68 μM). Of note, α‐triazolylboronic acids can be easily synthesized by click chemistry from readily available precursors, and with little purification issues. This could provide easy access to several possible analogues. In summary, we present α‐triazolylboronic acids as a promising PKI chemotype for further development of compounds with improved biological activity.

## Experimental Section

### Synthesis

α‐Amidoboronic acids,[^27^, ^48^] β‐triazolylboronic acids,[^35^, ^49^] α‐sulfonamidoboronic acids^[29]^ and α‐triazolylboronic acids[^36^, ^37^, ^38^] were synthesized and characterized according to previously described procedures. The general synthesis of β ‐triazolylboronic acids is reported in Scheme 1. The azidomethaneboronate (**1**), bearing the boronic moiety protected as (+)‐pinanediol ester, was allowed to react with the selected terminal alkyne **2** in a CuAAC reaction to obtain the (+)‐pinanediol α‐triazolylboronate **3**. Then the deprotection by transesterification with isobutylboronic acid allowed to obtain the compounds **4** in good overall yield (60–85 %).

### Synthesis of Compound 5 e and 6 e

#### 2‐(4‐(4‐aminophenyl)‐1*H*‐1,2,3‐triazol‐1‐yl)acetic acid (5 e)

Azidoacetic acid (37 μL; 0.49 mmol), NaAsc (19 mg; 0.098 mmol), and an aqueous solution of CuSO_4_ (0.98 mL, 0.03 M) were added to a solution of 3‐ethynylaniline (54 μL; 0.52 mmol) in *t*‐BuOH (1 mL). The reaction mixture was stirred for 2 hours at 60 °C under argon atmosphere. The mixture was then cooled to room temperature, diluted with EtOAc (10 mL×3) and extracted with a 1 : 1 H_2_O/brine (10 mL). Allowing the aqueous phase to stand overnight leads to the precipitation of the desired product **5 e** (25 mg; 23 %).

^1^H‐NMR (400 MHz, DMSO‐*d6*) δ 5.23 (s, 2H, CH_2_), 6.55 (d, *J=*8.5 Hz, 1H, aromatic), 6.96 (d, *J=*7.5 Hz, 1H, aromatic), 7.17–7.01 (m, 2H, aromatic), 8.33 (s, 1H, triazole).

^13^C‐NMR (101 MHz, DMSO) δ 46.28, 71.39, 111.30, 114.08, 114.67, 122.95, 130.10, 131.39, 147.49, 149.00.

ESI‐MS (m/z): theor. 219.09 [C_10_H_10_N_4_O_2_+H]^+^, exp. 219.05 09 [C_10_H_10_N_4_O_2_+H]^+^.

#### 4‐(1‐butyl‐1*H*‐1,2,3‐triazol‐4‐yl)aniline (6 e)

3‐Ethynylaniline (38 μL; 3.6 mmol) and bromobutane (39 μL; 3.6 mmol) were dissolved in *t*‐BuOH (12 mL). Subsequently, NaN_3_ (355 mg; 5.55 mmol), an aqueous solution of CuSO_4_ (12 mL; 0.03 M) and NaAsc (143 mg; 0.72 mmol) were added to the mixture. The reaction mixture was stirred for 2 hours at 60 °C under argon atmosphere. The mixture was then cooled to room temperature, diluted with EtOAc (15 mL×3) and washed with a 1 : 1 H_2_O/brine (15 mL). The organic phase was dried over Na_2_SO_4_, filtered and dried under reduced pressure. The resulting crude was further purified by column chromatography (silica gel, DCM/MeOH 98 : 2), leading to the isolation of **6 e** (216 mg; 28 %).

^1^H‐NMR (400 MHz, CDCl_3_) *δ* 0.96 (t, *J*=7.4 Hz, 3H, CH_3_), 1.32‐1.46 (m, 2H, CH_2_), 1.79‐1.99 (m, 2H, CH_2_), 4.37 (t, *J*=7.2 Hz, 2H, CH_2_N), 6.67 (m, 1H, aromatic), 7.10‐7.23 (m, 2H, aromatic), 7.27 (t, *J*=2.0 Hz, 1H, aromatic), 7.69 (s, 1H, triazole).

^13^C‐NMR (101 MHz, CDCl_3_) *δ* 13.59, 19.83, 32.42, 50.24, 112.54, 115.17, 116.32, 119.62, 129.87,

131.74, 146.70, 147.89.

ESI‐MS (m/z): theor. 217.14 [C_12_H_16_N_4_+H]^+^, exp. 217.13 [C_12_H_16_N_4_+H]^+^.

### *In Silico* Docking Studies

Molecular docking was performed using the Glide programme within the Maestro 13.4 Molecular modeling Suite from Schrödinger, LLC, New York, NY. The X‐ray crystal structure of FLT3 was obtained from the RCSB PDB databank https://www.rcsb.org (PDB code 4RT7). The protein structure was prepared according to the standard protein preparation workflow of Maestro, which includes adding hydrogen atoms, completing the missing loops, and assigning force field parameters. The grid was generated using the Receptor Grid Generation module using the centroid of the co‐crystallized ligand as center of the grid box; the box was set as a cube with a 10 A side. The docked compounds were prepared using the LigPrep module of Maestro using the PLS‐2004 force field to generate ionization states at pH 7.0±2.0, and multiple conformers of each compound. All molecules were then docked using XP precision, and the results visualised with pymol (www.pymol.org).

### Biochemical and Cellular Assays

In vitro kinase assays and cell viability assays were performed as described (Mologni et al., 2022).^[50]^ Briefly, recombinant His‐tagged FLT3 kinase domain (aa 569–992) was expressed in Sf9 cells and purified by nickel ion affinity chromatography. The purified enzyme was employed in an ELISA‐based kinase assay with 300 μM ATP and a Tyr‐phosphorylatable substrate peptide, using an anti‐phosphotyrosine antibody for detection (PY20; Santa Cruz Biotech). Cell viability was determined by MTS assay using the CellTiter 96® AQueous One Solution Cell Proliferation Assay kit (Promega) according to manufacturer's instructions. Dose‐response curves were calculated by non‐linear regression (log[inhibitor] *vs* normalized response) using GraphPad Prism software.

## Conflict of Interests

The authors declare no conflict of interest.

1

## Supporting information

As a service to our authors and readers, this journal provides supporting information supplied by the authors. Such materials are peer reviewed and may be re‐organized for online delivery, but are not copy‐edited or typeset. Technical support issues arising from supporting information (other than missing files) should be addressed to the authors.

Supporting Information

## Data Availability

The data that support the findings of this study are available from the corresponding author upon reasonable request.
