# Supplementary material for: α‐Triazolylboronic Acids: A Novel Scaffold to Target FLT3 in AML
Source: ChemMedChem. 2024 Nov 9;20(1):e202400622. doi: 10.1002/cmdc.202400622 (PMC11694611; doi:10.1002/cmdc.202400622)
Supplement: Supplementary file 1 — Supporting Information [file CMDC-20-e202400622-s001.pdf]

ChemMedChem

Supporting Information

α-Triazolylboronic Acids: A Novel Scaffold to Target FLT3 in AML

Maria Luisa Introvigne, Lorenza Destro, Luca Mologni, Valentina Crippa, Paolo Zardi, Francesco Fini, Fabio Prati, Emilia Caselli,* and Alfonso Zambon*

Supplementary information pertaining to

α- Triazolylboronic acids: a novel scaffold for targeting FLT3 in AML

Introvigne, M.L.;[a] Destro, L.;[b] Mologni, L;[c] Crippa, V;[c] Zardi, P.;[b] Fini, F.;[a] L.; Prati, F.;[a] Caselli, E.[a]* and Zambon, A.[b]*

a Department of Life Sciences, University of Modena and Reggio Emilia, Modena, Italy

b Department of Chemical and Geological Sciences, University of Modena and Reggio Emilia, Modena, Italy

c Department of Medicine and Surgery, University of Milano-Bicocca, Monza, Italy

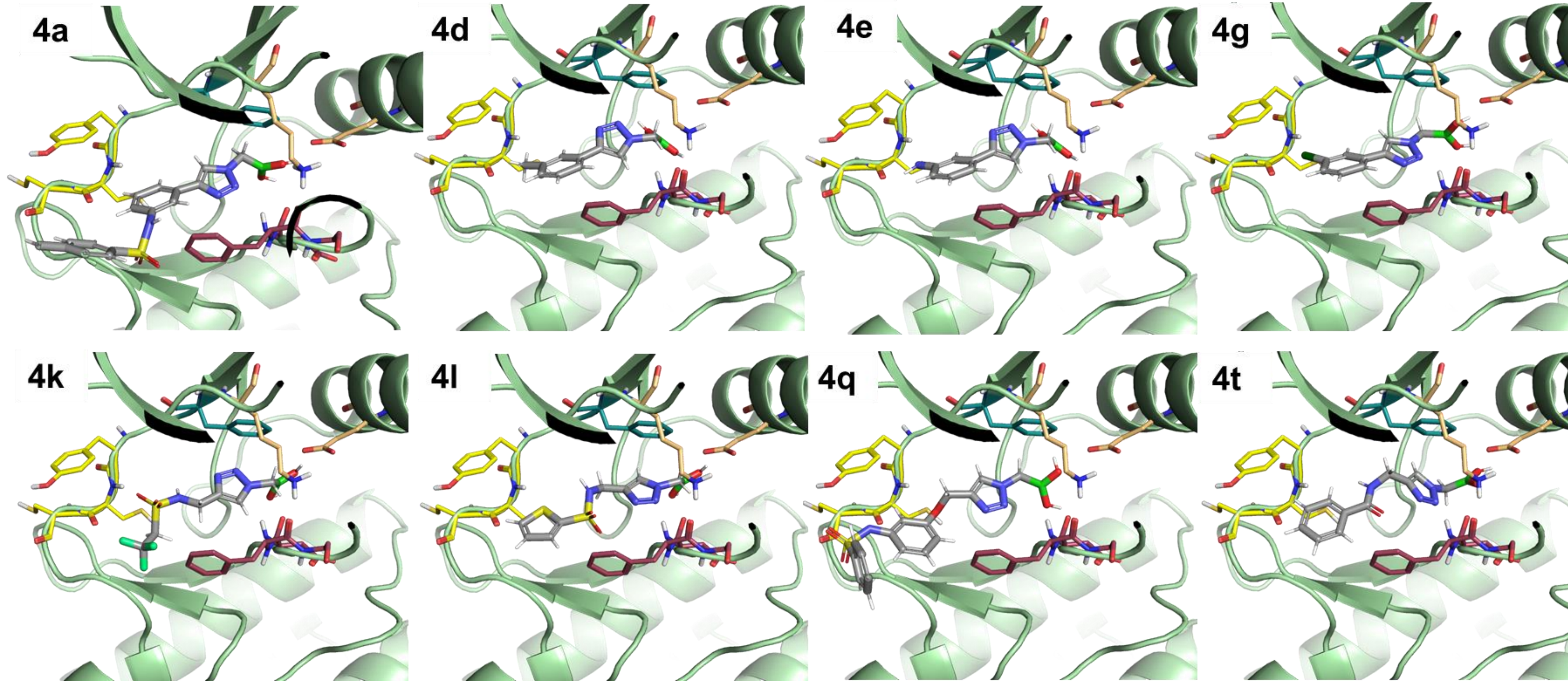

Supplementary Figure 1: docking pose of all active compounds on the co-crystal structure of FLT3 with quazartinib (PDB 4RT7)

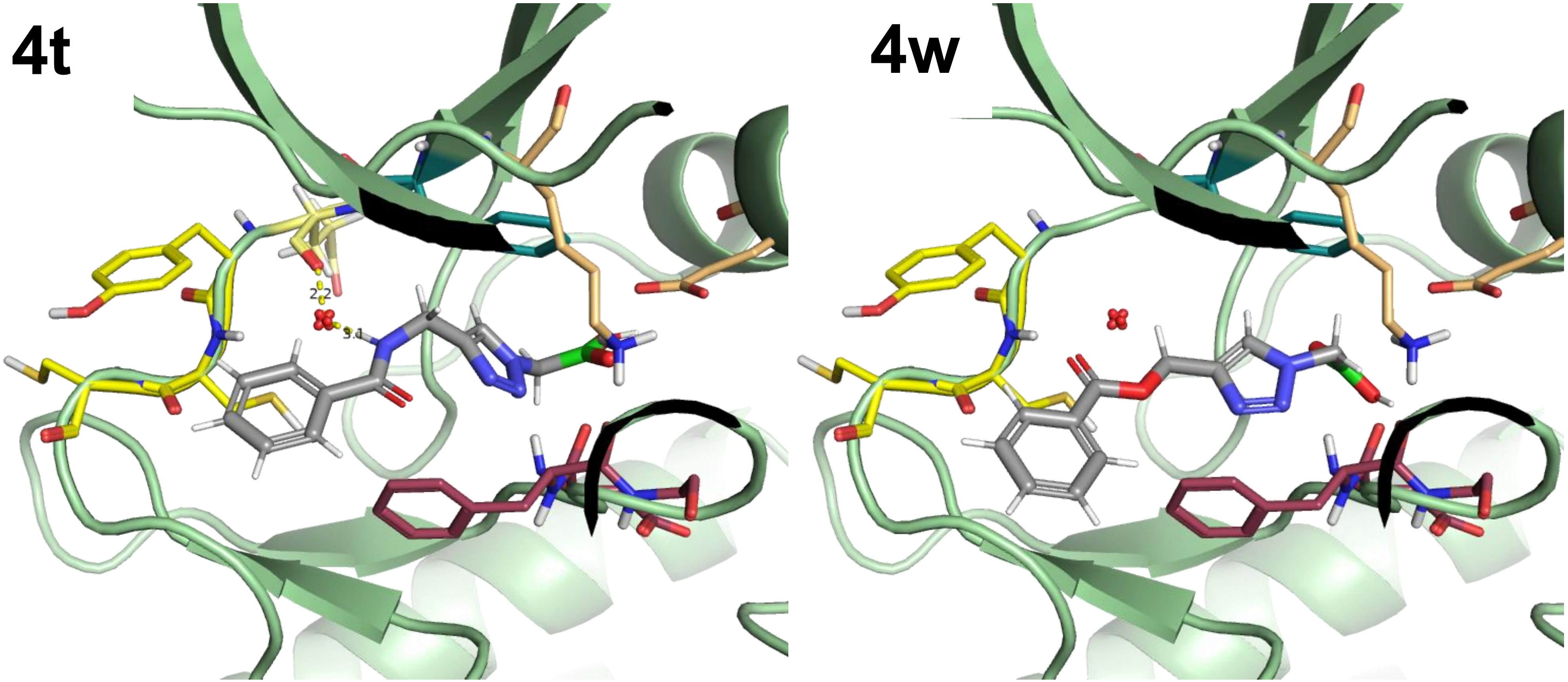

*Supplementary Figure 2: comparison of the docking pose of compound **4t** and **4w**:the Nh group of **4t** forms of a strong H-bond with a water molecule within the active site; NH-O replacement to ester **4w** impedes the interaction.*
